# Passive immunotherapy for Parkinson’s disease in 2025: Status and perspective

**DOI:** 10.4103/NRR.NRR-D-25-01792

**Published:** 2026-01-27

**Authors:** Alba González-Artero, Oriol Bárcenas, Jordi Pujols, Salvador Ventura

**Affiliations:** Institute of Biotechnology and Biomedicine (IBB) and Department of Biochemistry and Molecular Biology, Autonomous University of Barcelona, Bellaterra, Barcelona, Spain; Institute for Advanced Chemistry of Catalonia (IQAC) of the Spanish Council for Scientific Research (CSIC), Barcelona, Spain; Parc Taulí University Hospital, Parc Taulí Research and Innovation Institute (I3PT-CERCA), Autonomous University of Barcelona, Sabadell, Spain

Parkinson’s disease (PD) is the second most common neurodegenerative disorder after Alzheimer’s disease (AD). As a multisystem disorder, it presents with a complex and heterogeneous clinical profile (Kalia and Lang, 2015). The hallmark motor symptoms include bradykinesia, resting tremor, muscular rigidity, and postural instability. In addition, PD encompasses a wide spectrum of non-motor symptoms such as anosmia, rapid eye movement sleep behavior disorder, mood disturbances, and cognitive decline. As neurodegeneration progresses, patients experience a marked deterioration in quality of life, often resulting in long-term disability and a high degree of dependence on caregivers. Despite decades of research, available treatments for PD remain symptomatic, primarily aimed at restoring dopaminergic function or compensating for its loss. No disease-modifying therapies capable of halting or reversing neurodegeneration have yet been approved. This therapeutic gap has driven intensive efforts to develop novel strategies to block, slow, or reverse PD pathology. Among these, immunotherapeutic approaches have attracted increasing attention, inspired by recent advances in AD, with several monoclonal antibodies targeting amyloid-β now approved for clinical use.

PD pathogenesis is characterized by the irreversible accumulation of misfolded protein aggregates, a feature it shares with AD and other neurodegenerative disorders. In PD, intracellular inclusions, Lewy bodies and Lewy neurites, are primarily composed of α-synuclein (α-syn), a 14 kDa protein abundantly expressed at presynaptic terminals (Koga et al., 2021). In its native monomeric form, α-syn is soluble and intrinsically disordered, contributing to synaptic vesicle regulation and neurotransmitter release. Under pathological conditions, however, α-syn can misfold, oligomerize, and assemble into amyloid fibrils. Misfolded α-syn species are thought to seed the aggregation of native α-syn in neighboring neurons, propagating pathology through prion-like mechanisms across anatomically connected brain regions. These inclusions predominantly affect dopaminergic neurons in the substantia nigra pars compacta, resulting in progressive cell loss and dopamine depletion in the striatum. Although mature amyloid fibrils constitute the main structural components of Lewy bodies and Lewy neurites, intermediate species in the α-syn pathway, as oligomers, are increasingly recognized as the principal neurotoxic species driving cellular dysfunction and degeneration (Andrews et al., 2025). The accumulation of α-syn aggregates can occur in different cell types and brain regions, triggering a range of human diseases, including multiple system atrophy (MSA) and dementia with Lewy bodies. In MSA, oligodendrocyte survival is compromised by α-syn accumulation in glial cytoplasmic inclusions, whereas cortical and limbic neurons are the main targets of degeneration in dementia with Lewy bodies.

**Figure 1:**
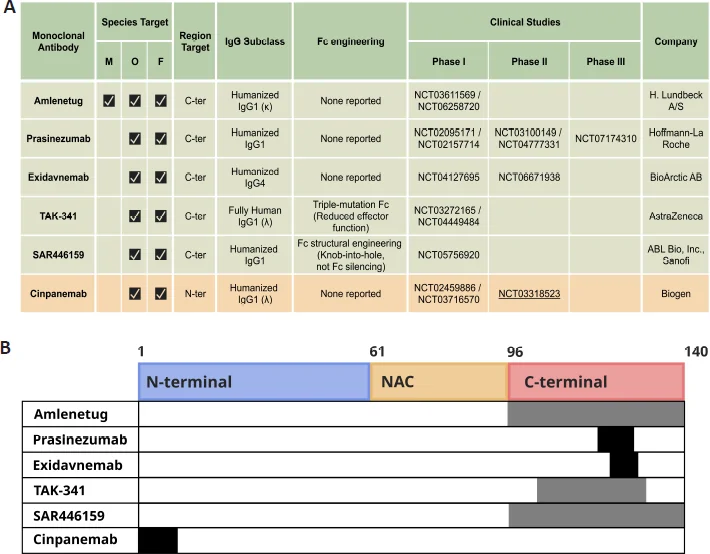
Summary of the landscape of therapeutic antibodies currently under development. (A) Overview of monoclonal antibodies developed for Parkinson’s disease. Conformational state refers to monomers (M), oligomers (O), and amyloid fibrils (F). Cinpanemab, the only antibody discontinued, is highlighted in orange, with the clinical trial that led to its discontinuation underlined. (B) Schematic of α-synuclein showing antibody epitope regions targeted by therapeutic candidates for Parkinson’s disease. Black bars indicate precisely mapped amino acid targets; grey bars indicate known binding regions without precise epitope definition. Created with Inkscape (version 1.2). NAC: Non-amyloid-beta component.

Given the central role of α-syn aggregation and propagation in PD pathogenesis and other synucleopathies, targeting these pathogenic species represents a rational therapeutic strategy. Antibody-based interventions aim to neutralize extracellular α-syn, prevent its intercellular spread, and facilitate its clearance by immune mechanisms. Building on insights from AD immunotherapy, several monoclonal antibodies are now advancing to clinical evaluation for PD. The following section reviews the landscape of these antibody candidates, their clinical outcomes, and the challenges that have shaped the field so far (**Figure 1**).

**Landscape of therapeutic antibodies under development:** Several monoclonal antibodies targeting α-syn are currently under clinical evaluation for PD and related synucleinopathies. Because antibodies primarily act in the extracellular space due to limited intracellular penetration, therapeutic strategies focus largely on neutralizing extracellular α-syn species and restricting their intercellular spread. Most candidates are designed to preferentially recognize aggregated or misfolded forms of α-syn, both oligomeric and fibrillar species, while minimizing engagement with the native monomeric protein to preserve its physiological role in synaptic regulation. The C-terminal region of α-syn has been identified as a key immunogenic target because it is exposed in pathological conformations and is accessible to antibody binding. Nonetheless, some antibodies aim for a broader recognition profile, including monomeric α-syn species, to maximize clearance potential; however, such strategies must carefully balance target engagement with the preservation of normal α-syn function. Amlenetug (LU AF82422), a human recombinant IgG1 monoclonal antibody developed by Genmab A/S and Lundbeck, exemplifies this strategy. It binds to the C-terminal region of α-syn and recognizes both monomeric and aggregated forms. Preclinical studies demonstrated that Amlenetug binding is largely confined to nervous tissue, minimizing off-target effects. In cynomolgus monkeys and mice, treatment reduced free plasma α-syn levels without inducing adverse effects, evidencing target engagement (Fjord-Larsen et al., 2021). Amlenetug’s safety for the treatment of PD has been evaluated in two different Phase 1 clinical trials (NCT06258720 and NCT03611569). However, following its FDA Fast Track designation as a potential therapy for MSA, current efforts are focused on the ongoing Phase 3 MASCOT trial, reflecting Amlenetug’s potential as a therapy for synucleopathies.

In contrast, most antibodies in advanced development selectively target aggregated α-syn. Prasinezumab, Exidavnemab, TAK-341, and SAR446159 are representative of this class. All four bind epitopes within the highly immunogenic C-terminal region. Prasinezumab, developed by Roche and Prothena, is a humanized monoclonal antibody derived from the murine precursor 9E4. In transgenic mouse models, 9E4 reduced α-syn pathology, improved motor and cognitive performance, and decreased neuroinflammation. In the phase II PASADENA trial, Prasinezumab showed signals of slowing motor symptom progression in some patients (Pagano et al., 2024). Although the subsequent Phase IIb PADOVA study failed to meet its primary endpoint, long-term exploratory analyses suggested slower progression in specific motor domains in subsets of early-stage patients. Under this statement, Roche recently initiated PARAISO (NCT07174310), the first Phase 3 clinical trial for a PD disease-modifying therapy. Exidavnemab (ABBV-0805), developed by BioArctic AB in collaboration with AbbVie, also targets the C-terminal region of α-syn. Its murine precursor, mAb47, displayed high selectivity for aggregated species and effectively reduced pathological accumulation in mouse models. A first-in-human study confirmed a favorable safety and pharmacokinetic profile (Nordström et al., 2021). Exidavnemab has since advanced to a Phase 2a clinical trial EXIST (NCT06671938), which will further assess biomarkers and its clinical efficacy in patients with PD and MSA. TAK-341 (MEDI1341), jointly developed by AstraZeneca and Takeda, is another C-terminal–binding antibody with high affinity and strong selectivity for aggregated α-syn. Preclinical studies demonstrated robust inhibition of pathology propagation in both *in vitro* and *in vivo* models (Schofield et al., 2019). Clinical testing has included a single-ascending dose study in healthy volunteers and a multiple-ascending dose study in PD patients (NCT04449484). TAK-341 is now being evaluated in a Phase 2 study in MSA. SAR446159, developed by Sanofi, is a bispecific monoclonal antibody engineered with an insulin-like growth factor 1 receptor-binding domain from ABL Bio, which acts as a brain-shuttle module to enhance blood–brain barrier translocation. It preferentially binds aggregated α-syn and inhibits its seeding activity both *in vitro* and *in vivo*. In aged mouse models overexpressing human α-syn, it reduced the spread of pathological species, provided neuroprotection, and improved motor performance. In nonhuman primates, antibody delivery to the cerebrospinal fluid and brain is enhanced compared to the parental antibody (An et al., 2025). A Phase 1 clinical trial evaluating its safety in healthy adults has been completed (NCT05756920).

An exception to the predominant C-terminal targeting strategy is Cinpanemab (BIIB054), developed by Biogen and Neurimmune. Cinpanemab binds the N-terminal region of α-syn, specifically the first 10 amino acids, with over 800-fold higher affinity for aggregated *versus* monomeric species. In preclinical studies, Cinpanemab reduced α-syn propagation and improved motor performance in mouse models. Despite promising preclinical activity, the antibody failed to meet primary and secondary endpoints in a Phase 2 trial (NCT03318523), leading to discontinuation of its development (Kuchimanchi et al., 2020).

Collectively, these clinical programmes illustrate the rapid evolution of antibody-based strategies for α-syn disorders. While preclinical and early clinical studies have consistently demonstrated safety, tolerability, and robust target engagement, the translation of these biological effects into measurable clinical benefit has not yet been confirmed for most of them. Of note, demonstrating antibody efficacy has proven far more complex than anticipated. Several factors likely contribute to the relatively high attrition observed in late-stage development, ranging from an incomplete understanding of mechanisms to methodological limitations within current clinical trial designs. The outcomes of these efforts will refine our understanding of PD pathology and its relationship with α-syn biology, trial design, and patient selection, laying the foundation for upcoming immunotherapeutic studies.

**Conclusion:** Based on recent clinical trial experience, it has become clear that translating biological activity into consistent clinical benefit remains a major challenge for α-syn-targeting immunotherapy. Although current antibodies have demonstrated encouraging pharmacokinetic, safety, and target engagement profiles, achieving measurable clinical efficacy has proven difficult. This reflects the complex interplay between PD neuropathology, the timing of therapeutic intervention, and the methodological constraints of current trial designs. Pathological α-syn aggregation begins many years before the onset of motor symptoms, meaning that most patients enrolled in trials already present substantial neuronal loss and extensive intracellular accumulation of aggregated α-syn. Because true disease modification is unlikely once degeneration is advanced, early, biomarker-driven recruitment is essential for maximizing therapeutic potential. The experience from the PADOVA and PASADENA studies reinforces this principle. Although the primary endpoints were not met in the initial study, analyses from the PASADENA open-label extension showed that prolonged treatment in early-stage PD was associated with slower motor progression. These findings underscore the need to align patient selection, early detection, treatment duration, and outcome measures more closely with the mechanisms of antibodies. A major bottleneck in PD drug development is the lack of reliable biomarkers, limiting early diagnosis, patient stratification, and assessment of therapeutic response. In contrast to AD, where cerebrospinal fluid phosphorylated Tau, Aβ_40_, and Aβ_42_ correlate with disease progression, neither total nor phosphorylated α-syn provides sufficient diagnostic accuracy in PD. Consequently, advances in fluid-based assays, molecular imaging, and digital monitoring are critical for tracking PD progression (Zarkali et al., 2024). Among emerging approaches, seed amplification assays are particularly promising, ultrasensitive tools for detecting α-syn amyloid aggregates. Seed amplification assays leverage the templating capacity of misfolded α-syn fibrils to amplify recombinant soluble protein *in vitro*, enabling detection from cerebrospinal fluid, skin, or mucosal samples and facilitating patient stratification. With detection limits in the femtogram range, seed amplification assays may identify individuals at risk prior to clinical diagnosis (Siderowf et al., 2023). Additional biomarker strategies include brain-derived extracellular vesicles and neurofilament light chain. Extracellular vesicles, present in blood and peripheral fluids such as saliva, carry neuronal proteins and nucleic acids, providing non-invasive access to PD-relevant markers, including extracellular vesicle-derived α-syn, allowing discrimination between PD, other synucleopathies, and healthy controls. Neurofilament light chain, detectable in blood and cerebrospinal fluid, reflects axonal damage and correlates with advanced disease stages, serving as a complementary marker of neurodegeneration. In parallel, imaging tools are needed. Notably, the positron emission tomography tracers ^18^F-F0502B and ^18^F-ACI-12589 exhibit α-syn selectivity, enabling *in vivo* visualization of α-syn pathology and its spatiotemporal progression (Smith et al., 2023; Xiang et al., 2023). Together, integrating the Unified Parkinson’s Disease Rating Scale with reliable molecular and imaging biomarkers will be critical to establishing a coherent clinical roadmap for PD drug development.

From a mechanistic perspective, targeting α-syn aggregation in the brain remains inherently challenging. Blood–brain barrier penetration is limited for large biomolecules, such as antibodies, necessitating the optimization of delivery to overcome poor biodistribution and suboptimal pharmacokinetic profiles. This constraint has driven the development of engineered delivery strategies. For example, SAR446159 incorporates an insulin-like growth factor 1 receptor–binding shuttle designed to enhance receptor-mediated transcytosis and brain exposure. Beyond insulin-like growth factor 1 receptor shuttles, multiple blood-brain barrier transport approaches have been explored preclinically, including bispecific receptor-mediated transcytosis platforms, Fc-engineering strategies to improve central nervous system uptake, and antibodies fused to transferrin receptor–binding domains, such as the oligomer-selective RmAb38E2-scFv8D3 antibody.

Optimization of antibody effector function is equally critical. IgG subclasses are favored due to their long serum half-life (**~**21 days) and defined immune activities. IgG1 is often selected for its strong ability to engage antibody-dependent cellular cytotoxicity and complement-dependent cytotoxicity, making it suitable for indications requiring robust immune activation, such as oncology or viral infections. In contrast, IgG4 exhibits minimal Fc receptor and complement engagement, rendering it more appropriate for chronic diseases where immune activation may be detrimental. Alternatively, IgG1 effector function can be silenced through Fc engineering. Common approaches include LALA mutations (L234A/L235A), which reduce Fc receptor binding, and N297 mutations (e.g., N297A, N297G), which eliminate Fc glycosylation and effector activity. Preclinical data indicate that effector-silent antibodies may reduce protein aggregation and mitigate cognitive deficits and neuropathology without inducing neuronal damage, supporting their potential for long-term treatment. However, recent evidence from Lecanemab (IgG1) underscores the importance of microglia-mediated amyloid plaque clearance, showing that this antibody has superior efficacy compared with engineered variants lacking immune effector function (Albertini et al., 2025).

In addition, the heterogeneity and dynamicity of α-syn conformers complicate effective target engagement and rational therapeutic design. Epitope selection plays a decisive role. C-terminal–binding antibodies appear more promising than earlier N-terminal approaches, as they preferentially recognize aggregation-prone and post-translationally modified species. Similarly, antibodies that preferentially target oligomeric or protofibrillar α-syn rather than its abundant functional monomeric form may exhibit greater apparent affinity for pathogenic conformers while minimizing interference with normal synaptic function. Selecting the optimal therapeutic target is therefore critical. Antibodies are thought to neutralize extracellular α-syn aggregates, restrict their prion-like spread, and promote microglial clearance. Of note, α-syn aggregates can persist within lysosomes or transfer between cells via tunnelling nanotubes. Clarifying the intercellular dynamics of α-syn transmission, including the mechanisms underlying aggregate trafficking, release, uptake, and long-range neurospreading, will be essential for improving the therapeutic efficacy of antibody-based strategies. Lessons from AD, particularly the success of lecanemab in selectively targeting soluble Aβ protofibrils, demonstrate that early and highly specific engagement of small, toxic aggregates can produce the greatest clinical impact. In PD, a comparable emphasis on soluble α-syn oligomers, together with enhanced central nervous system-delivery approaches and the recruitment of prodromal or at-risk individuals, may substantially increase the likelihood of therapeutic benefit.

In summary, the field of α-syn immunotherapy has advanced rapidly, with several antibodies now progressing through clinical development. Although significant challenges remain, the knowledge gained from recent clinical programs has ushered the field into a phase of methodological refinement, where trial design, patient selection, and biomarker strategies can be more effectively aligned with antibody mechanisms. These ongoing efforts are translating biological understanding into actionable parameters that inform next-generation studies. Ultimately, optimized epitope targeting, improved central nervous system delivery, and biomarker-guided stratification of prodromal and early-stage patients will determine whether antibody-based approaches can meaningfully alter the course of PD and other α-syn-driven disorders.
